# Key Considerations in Targeted Protein Degradation Drug Discovery and Development

**DOI:** 10.3389/fchem.2022.934337

**Published:** 2022-08-01

**Authors:** Liena Qin, Han Dai, Junfeng Wang

**Affiliations:** ^1^ Insilico Medicine Ltd., Shanghai, China; ^2^ High Magnetic Field Laboratory, CAS Key Laboratory of High Magnetic Field and Ion Beam Physical Biology, Hefei Institutes of Physical Science, Chinese Academy of Sciences, Hefei, China; ^3^ International Magnetobiology Frontier Research Center, Hefei, China; ^4^ Institute of Physical Science and Information Technology, Anhui University, Hefei, China

**Keywords:** targeted protein degradation, PROTAC, molecular glue, chemically induced proximity, drug discovery and development

## Abstract

Targeting proteins’ enzymatic functions with small molecule inhibitors, as well as functions of receptor proteins with small-molecule agonists and antagonists, were the major forms of small-molecule drug development. These small-molecule modulators are based on a conventional occupancy-driven pharmacological approach. For proteome space traditionally considered undruggable by small-molecule modulators, such as enzymes with scaffolding functions, transcription factors, and proteins that lack well-defined binding pockets for small molecules, targeted protein degraders offer the opportunity to drug the proteome with an event-driven pharmacological approach. A degrader molecule, either PROTAC or molecular glue, brings the protein of interest (POI) and E3 ubiquitin ligase in close proximity and engages the ubiquitin-proteasome system (UPS), the cellular waste disposal system for the degradation of the POI. For the development of targeted protein degraders to meet therapeutic needs, several aspects will be considered, namely, the selective degradation of disease-causing proteins, the oral bioavailability of degraders beyond Lipinski’s rule of five (bRo5) scope, demands of new E3 ubiquitin ligases and molecular glue degraders, and drug resistance of the new drug modality. This review will illustrate several under-discussed key considerations in targeted protein degradation drug discovery and development: 1) the contributing factors for the selectivity of PROTAC molecules and the design of PROTACs to selectively degrade synergistic pathological proteins; 2) assay development in combination with a multi-omics approach for the identification of new E3 ligases and their corresponding ligands, as well as molecular glue degraders; 3) a molecular design to improve the oral bioavailability of bRo5 PROTACs, and 4) drug resistance of degraders.

## Introduction

**FIGURE 1:**
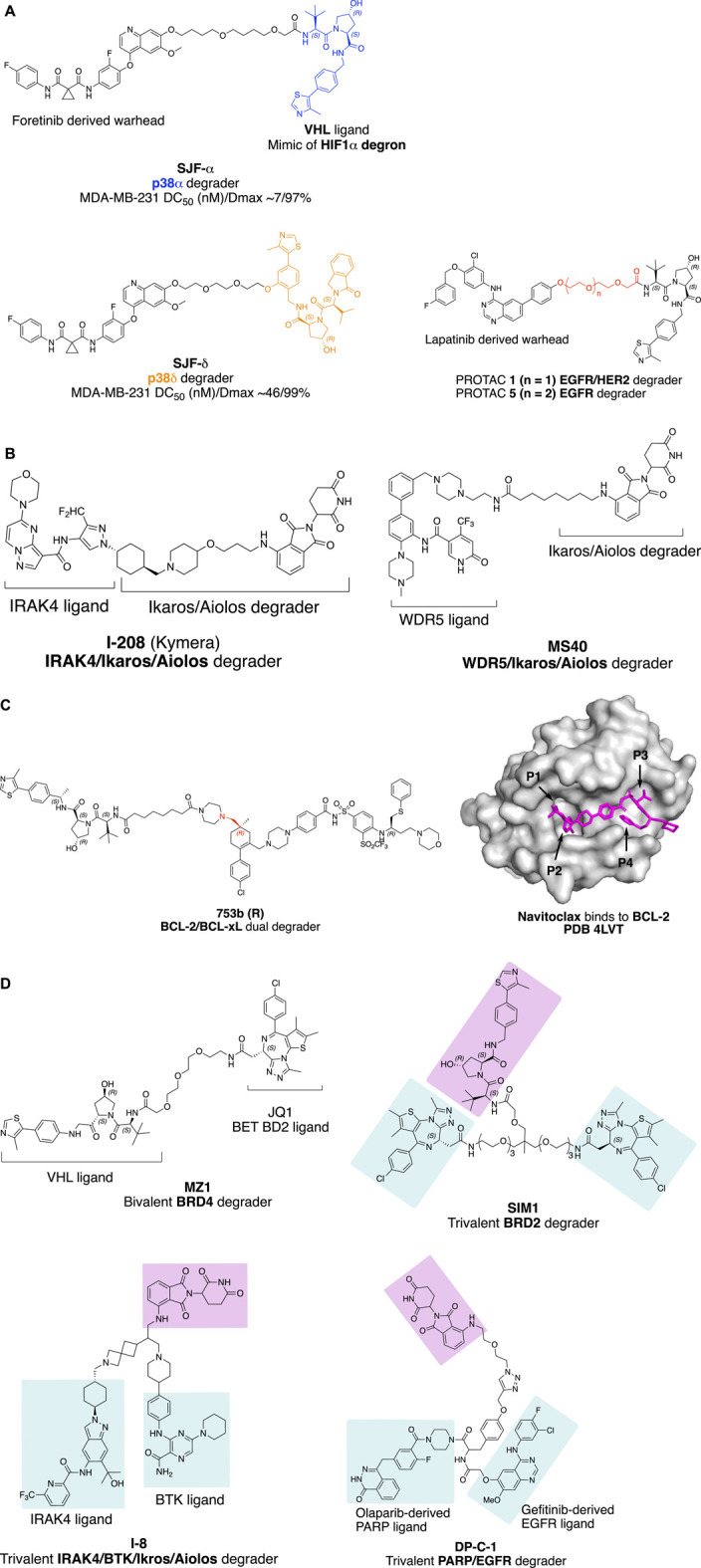
Factors influence the selectivity of degraders. **(A)** PROTAC with different linker attachment point to the VHL ligand selectively degrade p38α and p38β. **(B)** Structure of IRAK/Ikaros/Aiolos multi-targets degrader **I-208** and WDR5/Ikaros/Aiolos multi-targets degrader **MS40**. **(C)** Bcl-2/Bcl-xL dual degradation by **753bR** achieved with different linker attachment points to warhead; and crystal structure of Navitoclax with BCL-2 (Souers et al., 2013) with four pockets indicated with an arrow. The crystal structure was processed with PyMOL. **(D)** Trivalent PROTAC with multi-protein or multi-domain binding warheads. **MZ1** with a preference for BRD4 degradation, **SIM1** with a preference for BRD2 degradation.

PROTAC (proteolysis-targeting chimera) (Sakamoto et al., 2001) is a type of small molecule capable of the engaging ubiquitin-proteasome system, the cellular waste disposal system (Salami and Crews 2017), to degrade disease-causing proteins, by recruiting E3 ubiquitin ligase and labeling the target protein with polyubiquitin for proteasomal recognition. Classical PROTAC molecules are heterobifunctional small molecules consisting of two ligands connected with a flexible or rigid linker, with one ligand binding to POI, and the other binding to E3 ubiquitin ligase (Paiva and Crews 2019). The most prevalent E3 ligases used in pharmaceutical drug development are VHL (Von Hippel-Lindau) and CRBN (Cereblon) E3 ligases (Ishida and Ciulli 2021). Molecular glue degraders represented by immunomodulatory imide drugs (IMiDs) function similarly to PROTACs by engaging PPI (protein-protein interaction) between POI and E3 ligase, and directing the target protein for proteasomal degradation. Molecular glue (Schreiber 2021) degraders lack the typical linker seen in the PROTAC molecule. They are lower in molecular weight, and the binding affinity to each individual partner is lower or undetectable, as shown in the case of sulfonamide with DCAF15 and RBM39 (Du et al., 2019). In some cases, a degrader molecule could harness features of both PROTAC and molecular glue to degrade multiple targets (**I-208**, Figure 1B). In this review, both PROTAC and molecular glue approaches will be treated as small molecule targeted protein degraders.

Targeted protein degraders have the potential to target conventionally undruggable proteome (Samarasinghe and Crews 2021; Schneider and Chris, 2021), either as chemical biology research tools (Burslem and Crews 2020) or as new therapeutic modalities (Cromm and Crews 2017; Lai and Crews 2017; Nalawansha and Crews 2020), rapidly applied to cancer therapy (Dale et al., 2021), further applications include neurodegenerative disorders (Tomoshige and Ishikawa 2021). Pioneered by the AR (Androgen receptor) degrader **ARV-110** (NCT03888612) and ER (Estrogen receptor) degrader **ARV-471** (NCT04072952), developed by Arvinas Inc. for the treatment of prostate cancer and breast cancer, respectively, the field has seen at least 15 degraders in a clinical trial (Table 1) (Mullard 2021).

**TABLE 1:** Protein degraders approaching clinical trial (https://clinicaltrials.gov/) with structures disclosed.

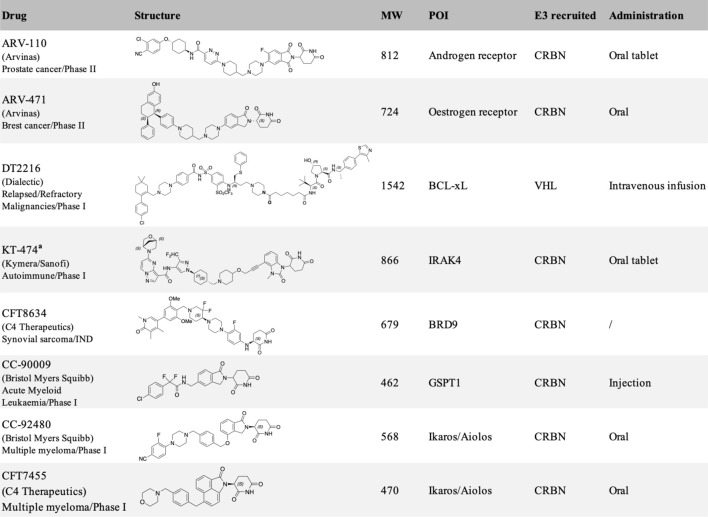

a Chemical structure of KT-474 was speculated from reported patent (Mainolfi et al., 2020).

Two aspects are key to fostering the development of targeted protein degraders in drug discovery and development, one is structure-guided design (Leissing, Luh, and Cromm 2020) of the heterobifunctional molecules, and the other is assay development driven by synthetic biology in combination with a multi-omics approach (Scholes et al., 2021) to systematically identify new E3 ubiquitin ligases and their corresponding ligands and molecular glue degraders. The design of selective PROTACs will be exemplified in the following context by the impact of linkerology on selective protein degradation, and the design of PROTACs to degrade multiple disease-causing proteins simultaneously. The impact of linkerology is also reflected in the physicochemical properties and oral bioavailability of PROTACs. Molecular design to improve oral bioavailability is important for bRo5 PROTAC drug development. The correlation of physicochemical features of PROTAC molecules with *in vivo* pharmacokinetics profile will be discussed. There are more than 600 E3 ubiquitin ligases encoded by the human genome, but only 2% of them have been applied in proximity-induced protein degradation. E3 ligases beyond VHL and CRBN for targeted protein degradation, for example, tissue-specific or disease-specific E3 ligases would considerably expand the application of targeted protein degradation for therapeutic purposes (Kannt and Đikić 2021; Guenette et al., 2022). Systematic searching for E3 ubiquitin ligases and their ligands is achieved using chemoproteomics methods applying cysteine reactive covalent small molecules to map the E3 ligase proteome. Assay development combined with multi-omics approaches is discussed. These benefit the targeted protein degradation field by providing the tools to systematically identify molecular glue degraders instead of being discovered by serendipity. At the end of the review, potential drug resistance mechanisms arising from targeted protein degradation will be briefly discussed. The design of degraders for therapeutic application discussed throughout the context reflects joint efforts from the chemistry and biology fronts to understand the molecular basis of disease pathways, the structure of productive ternary complex formation, as well as medicinal chemistry effort for the design of bioavailable molecules.

## Selectivity of Protein Degraders by Design

### Selective Degradation of Target Protein With Promiscuous Warhead

Turning a promiscuous small molecule inhibitor into a PROTAC, the selectivity could be rewired in the degradable proteome. A multi-kinase degrader generated by conjugating a highly promiscuous kinase inhibitor with CRBN-binding ligand was discovered to degrade a small set of kinases and CDK family proteins, using chemoproteomics method. In this study, Huang and coworkers demonstrated that target engagement is not sufficient for successful degradation (Huang et al., 2018). The selectivity of productive protein degradation is influenced by the E3 ubiquitin ligase (Lai et al., 2016), linker attachment points to the warhead, linker attachment point to E3 ligase ligand, and linker length (termed ‘linkerology’). The effect of E3 ubiquitin ligase selection and cell type on the degradation profile for PROTAC molecules will not be discussed here. This part will focus on the impact of linkerology on the selectivity of PROTAC molecules with promiscuous warheads.

The linker attachment point to a POI is usually selected at a solvent-exposed site of the warhead binding in a protein pocket. Linkers extended from a buried site may hinder the binding of a target protein and unsuccessful degradation. This can sometimes enhance the selectivity of a promiscuous warhead, as in the well-discussed case of enhancing the degradation of the cellular retinoic acid-binding protein (CRABP) over retinoic acid receptor (RAR) with the dual protein binder all-*trans* retinoic acid (ATRA) (Ishikawa et al., 2020).

Linker attachment point to E3 ligase ligand has the potential to influence the direction of assembly of the E3 ubiquitin complex. In a study evaluating protein degradation profile with promiscuous c-Met tyrosine kinase inhibitor Defactinib (Bondeson et al., 2018), p38α was found to be degraded (DC_50_ (nM)/D_max_ 210/91%) with VHL recruiting E3 ligase in triple-negative breast cancer cell line MDA-MB-231, while the MAPK family homolog p38δ, which shares 61% sequence identity with p38α, was only slightly degraded (∼30%). Later, a more potent selective p38α degrader **SJF-α** (MDA-MB-231 DC_50_ (nM)/D_max_ ∼7/97%) was developed (Smith et al., 2019). The linear linker and the VHL E3 ligase ligand were connected through the amide bond as depicted in Figure 1A. By changing the linker attachment point to the benzene ring of the VHL E3 ligase ligand through an ether bond, selective p38δ degradation (MDA-MB-231 DC50 (nM)/Dmax ∼46/99%) was achieved. The distinct degradation selectivity of two PROTAC molecules between two homologous MAPK family proteins was illustrated by *in vitro* stable ternary complex formation of VHL-**SJFα**-p38α and VHL-**SJFδ**-p38δ, respectively. Molecular dynamic simulation of the ternary complexes indicates that the VHL/p38α and VHL/p38δ interface was altered. VHL protein was recruited in a different direction approaching the p38α/p38δ protein due to the distinct linker attachment points of the PROTAC molecules.

The impact of linker length on the selectivity of PROTACs is exemplified by epidermal growth factor receptor (EGFR) and human epidermal growth factor receptor 2 (HER2) degrader with receptor tyrosine kinase inhibitor Lapatinib derived warhead (Burslem et al., 2018). PROTAC **1** with two PEG (polyethylene glycol) linker degrades both EGFR and HER2, while PROTAC 5 with three PEG linkers selectively degrade EGFR (Figure 1A). This type of exquisite selectivity is also reported in the CDK4/6 case with a highly conserved kinase active site (Anderson et al., 2020).

### Design of PROTAC Synergistically Degrade Multiple Disease-Causing Proteins to Meet the Clinical Needs

Interleukin-1 receptor-associated kinase 4 (IRAK4) is a serine/threonine-protein kinase with scaffolding functions, involved in Toll-like receptor (TLR, except for TLR3) and Interleukin-1 receptor (IL-1R) signaling pathways (Hennessy et al., 2010; Picard et al., 2011). IRAK4 is a 51KD protein that consists of an N-terminal Death Domain (DD residues 1–125), a hinge domain (residues 140–150), and a C-terminal kinase domain (residues 150–460). Upon TLR activation, IRAK4 is rapidly recruited by MYD88 to the receptor-signaling complex to form the Myddosome complex, then phosphorylates initially IRAK1 via oligomerization of the N-terminal DD in each of these proteins, leading to NF-κB nuclear translocation and activation. The scaffolding function of the DD of IRAK4 is essential in IL-1 signaling, while the kinase function of IRAK4 is partially responsible (Kim et al., 2007; De Nardo et al., 2018). To target both scaffolding function and kinase activity of IRAK4, degradation is superior over of IRAK4 kinase inhibitor, similar to that reported on FAK (Law et al., 2021). On the other hand, Ikaros and Aiolos are the activators of the redundant NF-κB pathway and upstream type I INF regulator. A PROTAC molecule capable of degrading IRAK4/Ikaros/Aiolos simultaneously could meet the clinical needs in treating B cell malignancy (Yang et al., 2012).

The key question to address for an IRAK4 degrader with Ikaros/Aiolos degradation properties is the selectivity. By proteomic analysis in thalidomide-treated H9 human embryonic stem cells, C2H2 zinc finger transcription factor SALL4 was identified as a *bona fide* neo-substrate of the thalidomide-CRBN-DDB1-Cul4A E3 ligase complex. Loss of function of SALL4 was verified to be responsible for thalidomide-caused teratogenicity (Donovan et al., 2018). Despite the side effects of thalidomide, it was later approved by FDA for the treatment of multiple myeloma under strict restrictions. Other thalidomide analogous immunomodulatory imide drugs (IMiDs), lenalidomide, and pomalidomide, have been developed for the treatment of cancer with fewer side effects and increased potency. Other than SALL4, IMiDs degrade a set of neo-substrates, most of which are C2H2 zinc finger proteins (Krönke et al., 2014; Donovan et al., 2018; Gao et al., 2020; Kozicka and Thomä, 2021) including Ikaros (IKZF1) and Aiolos (IKZF3). More selective Ikaros and Aiolos degraders are needed to reduce the safety concern as well as gain structural insights for the selectivity of degradation. **CC-92480** (Hansen et al., 2020) (NCT03989414) and **CFT7455** (Henderson et al., 2020) (Table 1) (NCT04756726) have been developed for the treatment of multiple myeloma and lymphoma by Bristol Myers Squibb and C4 Therapeutics independently, and are currently in phase I clinical trials. The selectivity of the IMiD degraders is achieved by introducing substitution to the phthalimide, therefore changing the approachable interface of CRBN by neo-substrates in the presence of IMiD (Leissing et al., 2020).

Kymera Therapeutics has designed a potent multi-targets IRAK4/Ikaros/Aiolos degrader **KT-413** for treating relapsed or refractory B-cell Non-Hodgkin’s lymphoma, which is in phase I clinical trial (NCT05233033). Although the structure of **KT-413** has not been disclosed, the molecule **I-208** (Figure 1B) (Mainolfi et al., 2020) has been disclosed to be able to induce *in vivo* degradation of IRAK4/Ikaros/Aiolos in OCI-LY10 tumor xenograft, correlated with tumor regression.

A similar approach of designing the PROTAC **MS40** (Figure 1B) for the degradation of MDR5/Ikaros with a synergistic effect in mixed-lineage leukemia (MLL)-rearranged leukemias was reported most recently (D. Li et al., 2022). WD repeat domain 5 (WDR5) is an integral component of histone lysine methyltransferase complex and MLL complex. MLL-rearranged leukemias also exhibit high expression and dependency on Ikaros. **MS40** was shown to degrade WDR5/Ikaros/Aiolos in acute lymphoblastic leukemia (ALL) RS4; 11 cells, and WDR5/Ikaros in biphnotypic B myelomonocytic leukemia MV-4–11cells at submicromolar range (MV-4-11 lack of expression of Aiolos). **MS40** has also shown modular *in vivo* tumor suppression activity in the subcutaneous MLL-AF9+AML PDX model, dosing at 100 mpk once daily for five days per week through intraperitoneal injection.

### Selective Degradation of Mono- or Bi-Target Protein With Dual Inhibitor

Navitoclax is a potent Bcl-xL and Bcl-2 dual inhibitor developed by AbbVie for the treatment of relapsed or refractory lymphoid malignancies. Navitoclax failed in the phase II clinical trial, due to on-target and dose-limiting thrombocytopenia (Mohamad Anuar et al., 2020). Platelets require Bcl-xL for survival. The VHL recruiting PROTAC **DT2216** (Table 1), utilizes Navitoclax as a warhead, achieved potent antitumor activity while less platelet toxicity *in vivo*, and is currently in Phase I clinical trial (NCT04886622). The reduced platelet toxicity was suggested to be due to the low expression level of VHL in platelet (He et al., 2019; Khan et al., 2019; He et al., 2020). The authors also validated that **DT2216** selectively degrades Bcl-xL in a Lys 87-dependent manner. Single Lys 87 mutation to arginine is sufficient to induce resistance to Bcl-xL degradation; if all the other lysines of Bcl-xL except Lys 87 were mutated, the degradation of Bcl-xL was retained.

The selective degradation of Bcl-xL over Bcl-2 could be explained by the linkerology of PROTAC molecule design. The linker of **DT2216** was designed by forming two amide bonds with VHL ligand and Navitoclax warhead respectively, one with the primary amine of the VHL ligand, and the other with the secondary amine of piperazine, which was converted from morpholine of Navitoclax. Linker extension from the morpholine binding site of Bcl-xL exposed the Lys 87 for ubiquitination, while Bcl-2 lacks such an accessible lysine, which results in the selective degradation of Bcl-xL over Bcl-2. The result is consistent with the finding that a productive ternary complex formation is required for targeted protein degradation (Chung et al., 2020).

Furthermore, degradation of both BCL-xL and BCL-2 with improved anti-leukemic activity was achieved by changing the linker attachment point to the methyl group, which is solvent-exposed located at pocket 1 (**P1**) and pocket 2 (**P2**) intersection of Bcl-2 or Bcl-xL as indicated in Figure 1C, to generate **753b** (R enantiomer) (Figure 1C) (D. Lv et al., 2021, 2). By doing so, the Lys 17 of Bcl-2 was accessible for ubiquitination according to the computational modeling of the Bcl-2-**753b**-VHL E3 ligase. Meanwhile, Lys 87 and Lys 20 of Bcl-xL remain accessible for ubiquitination in the BCL-xL-**753b**-VHL E3 ligase composition.

### Trivalent PROTAC With Bivalent Warhead

Trivalent PROTAC contains a bivalent warhead, which binds to two domains of one protein or two proteins simultaneously. Degrading dual-target proteins in the complimentary pathological pathway or synthetic lethal pair could be interesting to generate a synergistic effect in drug development. However, simultaneous degrading of such protein pair by a trivalent PROTAC requires the two proteins to be present at the same time and space in a cellular context. The design of a trivalent PROTAC requires careful planning of the linkerology, which has been well illustrated by the structure-guided design of a trivalent PROTAC with a warhead binding to two domains of one protein (Imaide et al., 2021). Bromodomain-containing proteins BRD2/3/4 and BRDT are members of the bromodomain and extra terminal domain (BET) family of proteins, structurally featuring two bromodomains (BD1 and BD2), which recognize acetylated lysine during transcriptional regulation. The well-known BET BD inhibitor **JQ1** (Filippakopoulos et al., 2010) was converted to **MZ1** (Zengerle et al., 2015) to give a VHL E3 ligase recruiting BET degrader. A more potent bivalent BD inhibitor **MT1,** which binds to the BD1 and BD2 of BET family proteins, was also reported with significant *in vivo* efficacy (Tanaka et al., 2016). Ciulli and coworkers validated the concept of developing a trivalent PROTAC **SIM1** (Figure 1D), guided by the chemical structure of **MZ1**, **MT1**, and the crystal structure of the BRD4 (BD2)-**MZ1**-VHL E3 ligase ternary complex (Gadd et al., 2017). The structure of BRD4 (BD2)-**MZ1**-VHL E3 ligase ternary complex suggests a three PEG point could be a branching point for another BD1 binding **JQ1** ligand. The eight PEG linker **SIM1** suggests sufficient length for linker attachment to the VHL ligand. The trivalent **SIM1** binds to both BD1 and BD2 domains of the BET protein and recruits VHL E3 ligase for targeted degradation of BET proteins. **SIM1** degrades BET family proteins with a preference for BRD2, which is different from **MZ1**’s preference for BRD4 degradation.

Trivalent PROTAC with a warhead targeting the synthetic lethal pair of EGFR and poly (ADP-ribose) polymerase (PARP) has been reported (Zheng et al., 2021). Based on the report that EGFR mutated lung cancer cells were sensitized to the treatment of PARP inhibitor Olaparib (Pfäffle et al., 2013), Zheng and coworkers designed a trivalent PROTAC with bivalent warhead derived from Olaparib and EGFR inhibitor Gefitinib (**DP-C-1**, Figure 1D). Both VHL and CRBN recruiting trivalent PROTACs were designed, and dose- and time-dependent degradation of EGFR and PARP was observed in non-small cell lung cancer cell line H1299 and pancreatic adenocarcinoma cell line SW1990 at μM range, respectively. Most recently, trivalent degraders targeting two synergistic protein targets, IRAK4 and BTK in B cell lymphoma, have been disclosed by Kymera Therapeutics (Weiss et al., 2022). The degraders represented by **I-8** (Figure 1D) also degrade Ikaros and Aiolos. Overall, these researches set a foundation for structure-guided design of PROTAC molecules for multidomain proteins, and potentially two protein targets synergistically for therapeutic benefits.

## Systematic Profiling of E3 Ligases, Ligands, and Molecular Glue Degraders

### Chemoproteomics Approach and Chemical Biology Assay Development for the Identification of New E3 Ligases and Ligands for Targeted Protein Degradation

The majority of PROTAC molecules in clinical trials recruit CRBN E3 ubiquitin ligase for targeted protein degradation (Table 1), including the AR degrader (**ARV-110**) (Crew et al., 2021), ER degrader (**ARV-471**) (Chen X. et al., 2021; Halford 2021), IRAK4 degrader (**KT-474**) (Mainolfi et al., 2020) (NCT04772885) and BRD9 degrader (**CFT8634**) (Nasveschuk et al., 2022) (NCT05355753). The Helios degrader, GSPT1 degrader (**CC-90009**) (Hansen et al., 2021) (NCT04336982), and Ikaros/Aiolos degraders are CRBN E3 recruiting molecular glue degraders. The only VHL E3 ligase recruiting PROTAC molecule currently in Phase I clinical trial is the Bcl-xL degrader (**DT2216**). Ligands of several E3 ubiquitin ligases including Nutlin-3a for MDM2 (Schneekloth et al., 2008) and Bestatin for cIAP (Itoh et al., 2010), have been reported for targeted protein degradation, and their application in drug development is still limited. The systematic approach for the identification of E3 ligases DCAF16 and RNF114 could be inspirational for the discovery of other new E3 ligases and their corresponding ligands (Spradlin et al., 2019; Zhang X. et al., 2019).

**FIGURE 2:**
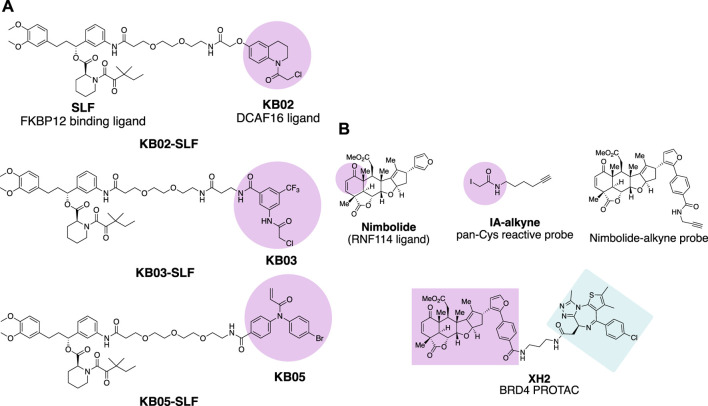
Chemical probes for the identification of E3 ligases. **(A)** FKBP12 degrading molecules with “scout” fragment **KB02, KB03,** and **KB05** covalently react with Cysteine in the proteome. **(B)** Chemical probes for the identification of E3 ligase recruited by **Nimbolide**.

Chloroacetamide and acrylamide containing “Scout” fragments are cysteine reactive electrophiles used by the pioneer of the Cravatt research team in activity-based protein profiling (Backus et al., 2016; Bar-PeledKemper et al., 2017). The ‘Scout’ fragments **KB02**, **KB03,** and **KB05** (Figure 2A) displayed broad cysteine reactivity in the human proteome, thus capable of capturing reactive cysteine residues of the E3 ubiquitin ligase pool once the scout fragments are turned into PROTAC molecules. Such PROTAC molecules are designed by linking FKBP12 binding protein-ligand SLF to the scout fragment. The molecules were tested in the HEK293 T cell line transduced with FLAG-tagged FKBP12 either with or without C-terminal NLS (nuclear localization sequence) KKKRKV. The compound **KB02-SLF** was found to promote the loss of nuclear FKBP12 in a Cullin E3 ligase and proteasome system-dependent manner. FLAG-mediated affinity enrichment was used to identify that the DCAF16 E3 ligase was associated with FKBP12_NLS degradation in a KB02-SLF-dependent manner (Zhang X. et al., 2019).

Nimbolide is a natural product derived from the Neem tree and possesses anticancer activity (Elumalai and Arunakaran 2014). The chemical structure of Nimbolide features an *α,β*-unsaturated ketone as Michael acceptor with the potential to act as an electrophile for the reactive cysteine residues of the target protein (Figure 2B). This enables isoTOP-ABPP (isotopic tandem orthogonal proteolysis-activity-based protein profiling) (Weerapana et al., 2010) to identify the direct protein targets of Nimbolide. The iodoacetamide-alkyne was used as the chemical probe in the experiment to react with those Cysteines spared by Nimbolide so that Nimbolide reactive cysteine-containing proteins will show differences in the quantitative mass spectrometry analysis. The E3 ligase RNF114 was identified to be the target of Nimbolide. The anticancer reactivity of Nimbolide arises from the inhibited ubiquitination and degradation of tumor suppressor p21 in the 231MFP breast cancer cell line by RNF114. The interaction of Nimbolide with RNF114 was further validated by pulldown of Flag-tagged RNF114 in 231MFP cells with the Nimbolide-alkyne probe (Figure 2B). The capability of E3 ligase RNF114 recruited by Nimbolide for targeted degradation was evaluated by the PROTAC molecule XH2 (Spradlin et al., 2019), to degrade BRD4 with the Bromodomain ligand **JQ1** as a warhead.

An indirect chemical biology method to evaluate the function of E3 ubiquitin ligases for targeted protein degradation is induced protein proximity by using a heterobifunctional small molecule (Ottis et al., 2017). E3 ubiquitin ligase and GFP were fused with HT7 (HaloTag7) and FKBP (FK506 binding protein) respectively, a heterobifunctional small molecule was designed with one side forming a covalent bond with Asp106 of HT7 while the other side binds to FKBP in a bump-hole mode. The E3 ubiquitin ligase and POI were induced close in space to evaluate the degradation signal. In the assay, GFP was used to give a fluorescent signal for monitoring the degradation event. More recently, the HiBit technology has been developed for measuring endogenous protein dynamics (Schwinn et al., 2020); and the NanoBRET assay (Riching et al., 2018) has also been developed to measure the kinetics of cellular degradation cascades. Those assays in combination provide methods to evaluate E3 ubiquitin ligases for targeted protein degradation in a high-throughput manner.

### Cell Biology Assay in Combination With Multi-Omics Methods for the Identification of Molecular Glue Degraders

**FIGURE 3:**
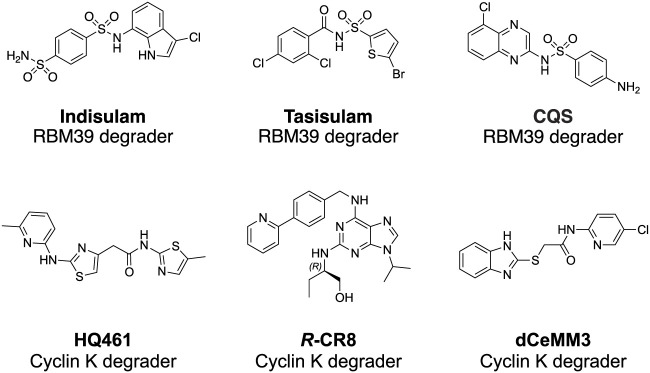
Structures of RBM39 degrading sulfonamides and CDK12 dependent Cyclin K degraders.

In the past, molecular glue degraders were usually found by serendipity while searching for the mode of action of small molecule drugs (Dong et al., 2021). Examples include thalidomide (Ito et al., 2010) and RBM39 (RNA binding motif protein 39) (T. Han et al., 2017) degrading sulfonamides (Figure 3) which were discovered to be molecular glue degraders. Thalidomide was used in the late 1950s and early 1960s for the treatment of morning sickness in pregnant women, which resulted in severe tragedies in causing thousands of miscarriages and birth defects. Via affinity-based protein profiling with a thalidomide-based probe in HeLa cell extracts, CRBN was found to bind to thalidomide, a substrate recognition subunit of DDB1-Cul4A Cullin RING E3 ubiquitin ligase. **Indisulam** was a small molecule compound with anticancer activity. The mode of action of indisulam was not revealed until recently by using a forward genetic method. Several single amino acid mutations of Indisulam were found in common across the RBM39 resistant HCT-116 cell line. RBM39 was later found to be degraded by indisulam in a dose-dependent manner. Following co-immunoprecipitation and liquid chromatography and mass spectrometry analysis, DCAF15 E3 ligase was found to be engaged in the degradation of RBM39.

Small molecules targeting protein homeostasis specifically by engaging protein-E3 ligase interactions for targeted degradation might be more common than previously known. Cell biology assays in combination with multi-omics methods have been developed for systematically searching for small molecules with such capability (Mayor-Ruiz et al., 2020; Scholes et al., 2021). Phenotypic screening of 2,000 cytotoxic compounds was carried out in WT and UBE2M (E2 enzyme) mutated (hyponeddylated) myeloid leukemia cell line KBM-7 to identify correlations between the toxicity of small molecules with the neddylation level. Since the neddylation of cullin-RING E3 ligases (CRLs) is highly associated with the E3s’ activity, the small molecules identified in the screen will be E3 ligase activity-dependent cytotoxic compounds. Four compounds (dCeMM1/2/3/4) were identified in the phenotypic screening. Quantitative expression proteomics revealed dCeMM1 to be RBM39 destabilizer and dCeMM2/3/4 to be a cyclin K degrader. CRISPR-Cas9 screening against all components of known CRLs revealed that cyclin K degradation is mediated by the CUL4B complex. Affinity-based protein profiling using a **dCeMM3**-derived chemical probe identified drug-mediated DDB1-CDK12-cyclin K complex formation. Drug sensitivity data for 4,518 clinical and pre-clinical compounds tested in 578 cancer cell lines were compared with the mRNA expression level of 499 E3 ligase components, and the cytotoxicity of the CDK inhibitor ***R*-CR8** (Figure 3) was found to correlate with the expression levels of CUL4 adaptor DDB1 (Słabicki et al., 2020). In the counter-confirmation experiment, sgRNA targeting *DDB1* conferred resistance to ***R*-CR8**. In the proteome-wide analysis of protein abundance following ***R*-CR8** treatment, cyclin K was the only protein shown to be consistently decreased. In the *in vitro* pulldown experiment of CDK12 (AA713-1052 kinase domain) bound cyclin K in the presence of ***R*-CR8**, DDB1 was significantly enriched versus in the absence of ***R*-CR8**. The crystal structure of CDK12^713−1052^-cycK^1−267^ bound to ***R*-CR8** and DDB1^ΔBPB^ was determined to illustrate the structural mechanism of ***R*-CR8** acting as a molecular glue degrader for cyclin K by strengthening the CDK12-DDB1 interaction.

At the same time, serendipitously, a screening effort for NRF2 inhibitors using NRF2 activity-based luciferase reporter assay, **HQ461** (Figure 3) was found to down-regulate NRF2 mRNA and expression levels (L. Lv et al., 2020). However, **HQ461**’s potent cytotoxicity in the A549 cell line was independent of NRF2 function. To explore the mechanism of function of the molecule, **HQ461** sensitive colorectal cancer cell line HCT-116 was treated with the compound to select resistant clones. Whole-exome sequencing against the **HQ461** resistant clones was performed to find the top-ranking variant was CDK12 G731E mutation. Both CDK12 and its interacting protein Cyclin K protein level were quantified, showing more than the 8-fold reduction of Cyclin K was observed after treatment with **HQ461** in the CDK12 wild-type cell line. The downregulation of Cyclin K was UPS-dependent. Pulldown using Flag-tagged CDK12 in the cell lysate treated with **HQ461** identified the interaction between CDK12 and DDB1. The **HQ461**-induced CDK12 (kinase domain)/CCNKΔC/DDB1 complex was further evaluated by AlphaScreen assay and chemical cross-linking mass spectrometry. The assay results give evidence that **HQ461** function as a molecular glue between CDK12 and DDB1, which triggers UPS-dependent depletion of Cyclin K.

## Improving the Oral Bioavailability of PROTAC Molecules

PROTAC molecules are usually beyond Lipinski’s rule of five (Ro5) (Caron et al., 2021) for orally administered drugs, conventionally considered to indicate poor permeability and oral bioavailability. A classical PROTAC molecule harnesses a warhead which is the small molecule ligand of the target protein, an E3 ligase ligand to recruit the VHL or CRBN subunit of Cullin ring E3 ligase, and a linker that brings the E3 ubiquitin ligase complex in close proximity to the target protein. Linkers of PROTAC molecules not only have a great influence on the degradation efficiency and selectivity of the target protein as previously reviewed (Cyrus et al., 2011; Zagidullin et al., 2020), but also had a profound impact on the *in vivo* PK profile of PROTACs, as shown in the cases of Androgen Receptor degraders and SMARCA2/4 degraders (Xiang et al., 2021; Kofink et al., 2022).

### Linker Rigidification to Improve the Oral Bioavailability of PROTAC

**FIGURE 4:**
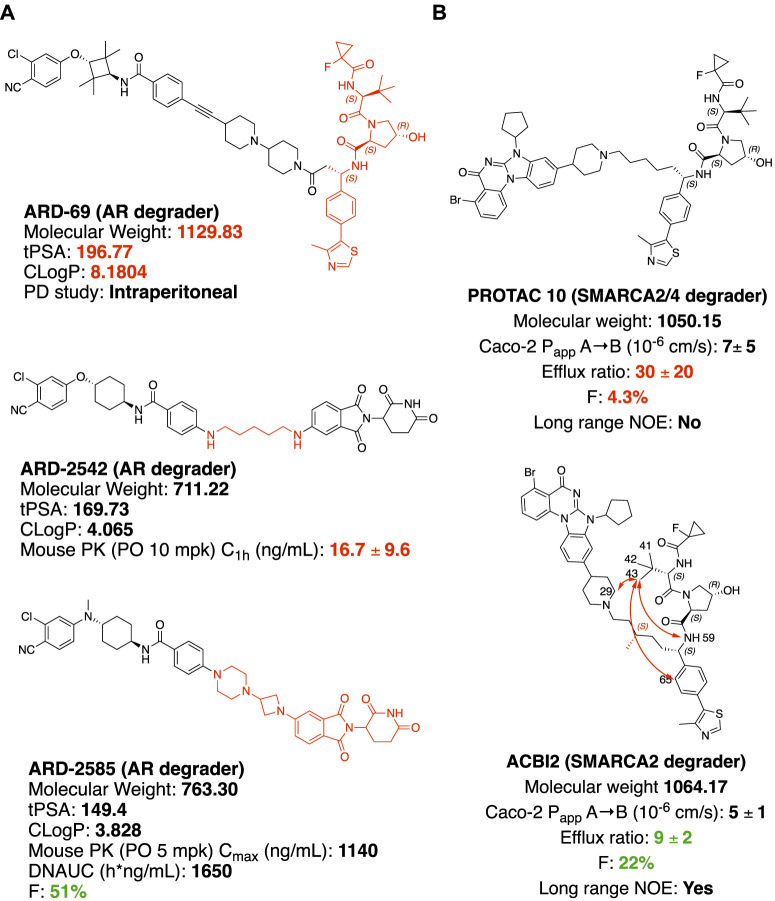
Structure modification to improve oral bioavailability. **(A)** Structures of AR degraders, ARD-69 with VHL ligand, ARD-2542 with a flexible linear linker, ARD-2585 with the rigid linker. **(B)** Structures of SMARCA2/4 degraders. Red arrows indicate atoms with long rang NOE.

In castration-resistant prostate cancer (CRPC), the progression of the disease is uncontrolled despite the low testosterone level, due to AR (androgen receptor) amplification and hypersensitivity, AR mutations, and intra-tumoral androgen production (Chandrasekar et al., 2015). In resistance development, AR antagonists could also be switched to agonists after treatment with inhibitors (Culig et al., 1999). Degradation offers new opportunities to tackle these problems with the event-driven pharmacological mechanism (Salami and Crews 2017). **ARV-110**, an orally available AR PROTAC developed by Arvinas Inc., is currently a Phase II clinical trial for the treatment of metastatic castration-resistant prostate cancer. With the success of **ARV-110**, Wang and coworkers achieved an orally available AR PROTAC by linker rigidification with a CRBN E3 recruiting ligand. **ARD-69** (Figure 4A) is a potent AR PROTAC with an enzalutamide analog as an AR binder and a rigid linker connected with the optimized VHL ligand (X. Han et al., 2019). **ARD-69**, with molecular weight >1000, calculated TPSA (topological polar surface area) = 197 and ClogP = 8 respectively, was administered intraperitoneal in the *in vivo* PD study. By changing the E3 recruiting element to thalidomide, the molecule **ARD-2542** induced efficient *in vitro* degradation of AR, since both VHL and CRBN are expressed ubiquitously and could induce efficient degradation of AR. Although with significantly reduced molecular weight, calculated TPSA and ClogP, **ARD-2542** exhibited a low plasma concentration of about 17 ng/ml after 1 h of oral administration at 10 mpk in a mouse pharmacokinetics study. Changing the linear linker to rigid piperazine and azetidines, **ARD-2582** plasma concentration in mice was increased with Cmax at 1140 ng/ml after oral administration at 5 mpk. The oral bioavailability increased to 51% in mice (Xiang et al., 2021).

### Solution Conformation of PROTAC Impacts the Permeability

SMARCA2 and SMARCA4 are close homologs as a component of the SWI/SNF complex, involved in chromatin remodeling and repair (Mashtalir et al., 2018; Chetty and Serra 2020). In SMARCA4-deficient cancer, selectively targeting SMARCA2 would be a potential synthetic lethal therapeutic method to treat cancer (Hoffman et al., 2014). SMARCA2 and SMARCA4 share 73.6% of protein sequence identity by EMBOSS Needle pairwise sequence alignment (Madeira et al., 2019), containing both ATP-dependent helicase domain and bromodomain (BD) domain. Small molecule inhibitors of the bromodomain developed so far inhibit the bromodomain of both SMARCA2 and SMARCA4 (Theodoulou et al., 2016). Additionally, the ATP-dependent helicase function of SMARCA2 is not targeted by the bromodomain inhibitor. An orally available SMARCA2 selective degrader would have potential therapeutic value over small molecule inhibitors. **ACBI2** (Figure 4B) was reported by Kofinik and coworkers to be SMARCA2 selective PROTAC with improved oral bioavailability over PROTAC **10** (Figure 4B) via a minor change of the linker (Kofink et al., 2022). The PROTAC **10**, with a five-carbon chain to link a SMARCA2/4 BD inhibitor and a VHL E3 ligase ligand, turned out to be a SMARCA2/4 degrader with only 4.3% oral bioavailability in mouse pharmacokinetic studies. The poor oral bioavailability was attributed to its poor permeability as indicated by the high efflux ratio from an *in vitro* Caco-2 permeability test. Introducing a methyl group to the full carbon chain to generate **ACBI2**, dramatically reduced the efflux ratio and thus increased the oral bioavailability to 22%. MD (Molecular Dynamics) simulation and NOE (Nuclear Overhauser effect) NMR spectroscopy were carried out to elucidate the link between conformational restraint and reduced efflux ratio. **ACBI2** was found to have reduced TPSA by MD simulation compared to PROTAC **10**. NOE is usually observed in NMR experiments between protons close in space. Long-range NOE observed in **ACBI2** but not PROTAC **10** indicates that **ACBI2** adopts a more constrained solution structure, which explains the reduced efflux ratio. For PROTAC with a constrained structure, macrocyclization could be a design strategy. In a case reported by Testa and coworkers, macrocyclization of **MZ1** leads to a 12-fold loss of binding to BRD4, however, the comparable cellular degradation activity to **MZ1** may indicate increased cell permeability (Testa et al., 2020). Because of the unique properties of bRo5 molecules, new descriptors such as EPSA and ChameLogD (Ermondi et al., 2020; Caron et al., 2021) have been introduced to take dynamic intramolecular hydrogen bond (dIMHB) into consideration for better correlation of the physicochemical properties of PROTACs with Caco-2 cell permeability profiles.

## Drug Resistance in Targeted Protein Degradation

One of the advantages of PROTAC is in overcoming some of the resistance mechanisms to traditional targeted therapies (Burke et al., 2022), represented by AR PROTAC ARV-110 to address metastatic castration-resistant prostate cancer (mCRPC) (Salami and Crews 2017; Halford, 2021; Mullard 2021). Acquired drug resistance quite often occurs during the use of clinical small molecule inhibitors or antagonists, such as T790M and C797S mutation of EGFR conferred drug resistance induced by EGFR inhibitors (Thress et al., 2015). Although the resistance could be addressed by developing third- or fourth generations of EGFR inhibitors, new drug resistance will emerge. PROTAC technology has shown certain advantages in overcoming drug resistance against cancer drug targets due to the degradation of target proteins with reduced evolutionary pressure of target mutations (Shibata et al., 2017; Burslem et al., 2018; Burslem et al., 2019; Flanagan et al., 2019; Cheng et al., 2020; Robbins et al., 2020; Liu et al., 2021; Robbins et al., 2021). However, new mechanisms of drug resistance may occur (Zhang L. et al., 2019). Several research teams have revealed the vulnerabilities of UPS using siRNA-based loss-of-function screening (Ottis et al., 2019), resistance mutations by CRISPR-suppressor scanning (Gosavi et al., 2022), and potential acquired resistance mechanism against degraders by whole-exome sequencing in degrader selected cells lines (Zhang L. et al., 2019). The study of acquired resistance was carried out in SKM1, MV4; 11, LNCaP, and OVCAR8 cell lines. Resistance cell lines were selected after 4 months of treatment with BET PROTACs. The two AML cell lines (SKM-1 and MV4; 11) and the prostate cancer cell line (LNCaP) were much more sensitive to the compounds’ treatment compared with the ovarian cancer cell line OVCAR8. No stable resistant clones were obtained from SKM-1, MV4; 11, and LNCaP cell lines. The resistant clones from the BET PROTAC insensitive OVCAR8 cell line were further validated. The genomic and transcriptional analysis indicated that resistance to VHL-based BET PROTAC was caused by CUL2 loss due to multiple genetic alterations at the CUL2 locus; the resistance to CRBN-based BET PROTAC was due to chromosomal *CRBN* gene deletion. PROTAC is usually applied to the cancer cell lines highly dependent on the UPS system for therapeutic purposes, therefore the probability of acquired resistance due to loss of function of E3 ubiquitin ligase is small. Although there is no reported PROTAC resistance in the clinic yet, with more degraders approaching clinical trial, it is important to look for new cancer cell line essential E3 ligases for precision medicine.

### Perspective

**FIGURE 5:**
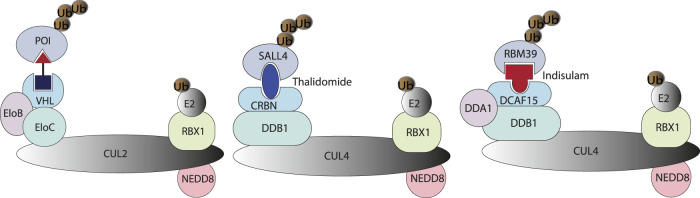
A PROTAC molecule is represented by a heterobifunctional molecule with a linker, which induces proximity between VHL and POI. Molecular glue degraders are represented by Thanlidomide and Indisulam-induced protein-protein interaction between neosubstrate and substrate-binding subunit of E3 ubiquitin ligase (Bulatov and Ciulli 2015).

In cells, DNA, RNA, and proteins are the key elements at the foundation of biological complexity, forming the backbone of what Francis Crick in 1957 termed the “Central Dogma” of molecular biology. However, according to Stuart Schreiber, there is a missing link in the network of Central Dogma: small molecules. Small molecules have critical roles at all levels of biological complexity and yet remain orphans of the Central Dogma (Schreiber 2005). Small molecule perturbation of protein functions has contributed a profound part to modern small molecule drug discovery (Beck et al., 2022). In addition to individual protein targeting by small molecules, chemically-induced proximity by heterobifunctional small molecules could redirect the biological processes of protein homeostasis. Targeted protein degrader is the type of induced-proximity molecule which targets POI for the posttranslational modification (PTM) of ubiquitination and subsequent proteasomal degradation (Figure 5). Protein homeostasis is regulated by many other types of PTMs (Uversky 2013), including but not limited to phosphorylation, acetylation, SUMOlyation, hydroxylation, farnesylation, glycosylation, and ADP-ribosylation. These PTMs of proteins regulate protein life span, protein cellular location, and protein function. Small molecules targeting the protein homeostasis by inducing PTM beyond ubiquitination may impact small molecule drug development in the pharmaceutical industry. PROTAC will offer the opportunity to target traditionally undruggable targets by an event-driven pharmacological approach, opening new therapeutic modalities (Békés et al., 2022) to expand the druggable space. Inspired by PROTAC, induced-proximity drug modalities including LYTAC (Banik et al., 2020), AUTAC (Takahashi et al., 2019), ATTEC (Z. Li et al., 2020), PhosTAC (Yamazoe et al., 2020; Chen P.-H. et al., 2021), DUBTAC (Kanner et al., 2020; Henning et al., 2021) and RIBOTAC (Haniff et al., 2020; P.; Zhang et al., 2021), are of interest to the pharmaceutical industry, allowing targeting of disease-causing proteins and even RNAs. This has resulted in the emergence of new start-up companies in the targeted protein degradation area (https://www.ventureradar.com/startup/Protein%20Degradation). The developments within Cryo-EM and X-ray crystallography technology, CRISPR-Cas technology-based assay development, and increasing sequencing capability will additionally strengthen the structure-guided design and multi-omics approach to designing small molecule therapeutics with induced-proximity mechanisms.
